# Draft genome sequences of three *Streptomyces* sp. strains isolated from marine sponges in Kochi Prefecture, Japan

**DOI:** 10.1128/mra.01479-25

**Published:** 2026-02-13

**Authors:** Aya Hirano, Yuta Matsubara, Kaito Okamura, Tetsuya Sakurai, Dana Ulanova

**Affiliations:** 1 Graduate School of Integrated Arts and Sciences, Agriculture and Marine Science, Kochi University 12888, Nankoku, Kochi, Japan; 2 Department of Marine Resource Science, Faculty of Agriculture and Marine Science, Kochi University 12888, Nankoku, Kochi, Japan; 3 Marine Core Research Institute, Kochi University, Nankoku, Kochi, Japan; Montana State University, Bozeman, Montana, USA

**Keywords:** *Streptomyces*, marine microbiology

## Abstract

We report the draft genome sequences of three actinobacteria, *Streptomyces* sp. strain A3, *Streptomyces* sp. strain C3, and *Streptomyces* sp. strain J1. These strains were isolated from three different marine sponge specimens and shared over 99.9% average nucleotide identity at the genome level.

## ANNOUNCEMENT

Marine-derived *Streptomyces* species present an untapped source of natural products, with antimicrobial and other bioactivities (1). During a survey of culturable streptomycetes and their bioactive compounds from marine sponges, we isolated three strains, *Streptomyces* sp. strain A3, *Streptomyces* sp. strain C3, and *Streptomyces* sp. strain J1, and present their draft genome assemblies in this report.

Three samples of marine sponge (unidentified Demospongiae) were collected by scuba diving offshore Kochi Prefecture, Japan (33°25′27.25″ N, 133°27′29.04″ E, water depth 10–15 m), and processed as described previously (2). The homogenized sponge samples were inoculated on AMM agar plates prepared using distilled water or seawater (3) and on actinomycete isolation agar (Difco) prepared using distilled water. Media were supplemented with rifampicin (5 mg/L) and/or cycloheximide (100 mg/L). The agar plates were cultivated at 28°C for 30 days, and actinomycete-like colonies were transferred to fresh media until pure cultures were obtained.

The isolates were cultured in AMM liquid medium (2 days, 28°C, 160 rpm), and genomic DNA was extracted using Genomic DNA Buffer Set and Genomic-Tip 20/G (both QIAGEN) according to the manufacturer’s protocol. The DNA quantity and quality were evaluated by a Qubit 3.0 fluorometer (Thermo Fisher) and a NanoVue Plus spectrophotometer (GE Healthcare).

The library preparation and sequencing were performed by Rhelixa, Inc., using the NEBNext Ultra II DNA Library Prep Kit and Illumina NovaSeq X Plus (150 bp × 2 paired end) according to the manufacturer’s protocols. The raw reads were trimmed using fastp version 1.0.1 (4) to remove adapter and low-quality sequences. The obtained reads were assembled by the SPAdes Assembler version 4.1.0 (5). Genome data were checked for contamination using the ContEst16S tool (6). The genome annotation was generated by the NCBI Prokaryotic Genome Annotation Pipeline (7). Prediction of biosynthetic gene clusters (BGCs) for bioactive compounds was done by antiSMASH bacterial version 8.0 (8). Average nucleotide identity (ANI) was calculated by ANI Calculator, EZ Bio Cloud (9). All tools except for fastp were run with default parameters. Fastp was run with -q 20 -w 16 --detect_adapter_for_pe options.

The isolation media, the number of raw reads, and details of data processing for each strain are shown in Table 1. Strains A3, C3, and J1 shared 99.9% average nucleotide identity at the genome level. All strains shared 100% 16S rRNA gene sequence identity (BLASTn [nucleotide collection database; 10]) and 98.8%–98.9% ANI with *Streptomyces* sp. SM17 (GenBank CP029338.1). *Streptomyces* sp. SM17, which produces an antifungal compound called surugamide (11), was obtained from a marine sponge, *Haliclona simulans*, from the west coast of Ireland (12). Similarly, antiSMASH analysis revealed the presence of a BGC homological to surugamide BGC in all three strains isolated in this study.

**TABLE 1:** Isolation media and sequencing data of *Streptomyces* sp. strains A3, C3, and J1

	*Streptomyces* sp. strain A3	*Streptomyces* sp. strain C3	*Streptomyces* sp. strain J1
Isolation medium	AMM agar (distilled water), cycloheximide, rifampicin	Actinomycete isolation agar, cycloheximide, rifampicin	Actinomycete isolation agar, cycloheximide
Raw reads	21,392,058	18,778,856	18,876,772
QC reads	20,813,324	18,278,214	18,375,040
Total assembly length (bp)	7,368,253	7,106,445	7,364,695
Contigs	277	199	233
Largest contig (bp)	229,085	312,672	223,552
N50 (bp)	67,558	78,836	61,367
Coverage	423.42	384.74	374.02
GC content	73.28	73.43	73.31
Genes (total)	6,461	6,214	6,456
CDSs (total)	6,379	6,134	6,375
CDSs (with protein)	6,283	6,056	6,279
tRNAs	67	66	68
rRNAs (5S, 16S, 23S)	5, 3, 4	5, 2, 4	4, 2, 4

*Streptomyces* species containing surugamide BGCs were reported from other marine and soil environments around the world (12). Our study adds a new location of surugamide BGC-containing streptomycetes and brings new insights on biogeography of *Streptomyces* and natural product BGCs.

## Data Availability

These Whole Genome Shotgun Projects have been deposited at DDBJ/ENA/GenBank under accession numbers JBSYHI000000000 (*Streptomyces* sp. A3), JBSYHH000000000 (*Streptomyces* sp. C3), and JBSYHG000000000 (*Streptomyces* sp. J1). The versions described in this paper are versions JBSYHI010000000 (*Streptomyces* sp. A3), JBSYHH010000000 (*Streptomyces* sp. C3), and JBSYHG010000000 (*Streptomyces* sp. J1). The raw reads are available in the Sequence Read Archive under BioProject with accession number PRJNA1375478.
